# A putative glucose 6-phosphate isomerase has pleiotropic functions on virulence and other mechanisms in *Acidovorax citrulli*

**DOI:** 10.3389/fpls.2023.1275438

**Published:** 2023-11-07

**Authors:** Lynn Heo, Yoobin Han, Yongmin Cho, Junhyeok Choi, Jeongwook Lee, Sang-Wook Han

**Affiliations:** Department of Plant Science and Technology, Chung-Ang University, Anseong, Republic of Korea

**Keywords:** Acidovorax citrulli, proteomics, glucose 6-phosphate isomerase, virulence, pleiotropic effect

## Abstract

*Acidovorax citrulli* (*Ac*) is a causal agent of watermelon bacterial fruit blotch (BFB) disease. Because resistance cultivars/lines have not yet been developed, it is imperative to elucidate *Ac*’s virulence factors and their mechanisms to develop resistant cultivars/lines in different crops, including watermelon. The glucose-6-phosphate isomerase (GPI) is a reversible enzyme in both glycolysis and gluconeogenesis pathways in living organisms. However, the functions of GPI are not characterized in *Ac*. In this study, we determined the roles of GpiAc (GPI in *Ac*) by proteomic and phenotypic analyses of the mutant lacking GPI. The mutant displayed significantly reduced virulence to watermelon in two different virulence assays. The mutant’s growth patterns were comparable to the wild-type strain in rich medium and M9 with glucose but not with fructose. The comparative proteome analysis markedly identified proteins related to virulence, motility, and cell wall/membrane/envelope. In the mutant, biofilm formation and twitching halo production were reduced. We further demonstrated that the mutant was less tolerant to osmotic stress and lysozyme treatment than the wild-type strain. Interestingly, the tolerance to alkali conditions was remarkably enhanced in the mutant. These results reveal that GpiAc is involved not only in virulence and glycolysis/gluconeogenesis but also in biofilm formation, twitching motility, and tolerance to diverse external stresses suggesting the pleiotropic roles of GpiAc in *Ac*. Our study provides fundamental and valuable information on the functions of previously uncharacterized glucose 6-phosphate isomerase and its virulence mechanism in *Ac*.

## Introduction

*Acidovorax citrulli* (*Ac*), formerly known as *A. avenae subsp. citrulli* is a Gram-negative, rod-shaped, aerobic, and seed-borne bacterium. It causes bacterial fruit blotch (BFB) disease in cucurbit crops, including watermelon worldwide (Schaad et al., 1978; Willems et al., 1992; Burdman and Walcott, 2012). *Ac* infection in watermelon occurs at any growth stage and in all plant tissues (Rahimi-Midani and Choi, 2020). At the initial growth stage, water-soaked lesions and wilt symptoms are observed on the cotyledons and seedlings, respectively (Burdman et al., 2005). When *Ac* infects the watermelon fruit, water-soaked lesions occur on the surface of the fruit, the lesions are turned black, and necrosis is observed in the internal parts of the fruit (Latin and Hopkins, 1995). *Ac* strains are classified into two groups (Zivanovic and Walcott, 2017); Group I is generally virulent to non-watermelon cucurbits, including melon, while Group II causes severe disease in watermelon. BFB disease poses a major threat to cucurbit crop production and industry worldwide (Shrestha et al., 2013; Islam et al., 2020). Despite the economic significance of the disease, the strategies to effectively manage BFB have not been elucidated in different crop species. In addition, watermelon cultivars resistant to the group II strains have not been developed yet. Therefore, it is essential to elucidate the virulence factors of *Ac* group I strain and its related mechanisms to design a strategy to control the disease.

Various virulence factors/mechanisms of *Ac* have been characterized. A previous study reported that *Ac* mutants with impaired twitching motility and biofilm formation were less virulent than the wild-type strain (Bahar et al., 2009). Type 4 pili, mediating twitching motility, are known to be involved in surface adhesion and virulence (Craig et al., 2004; Bahar et al., 2009; Yang et al., 2023). Biofilm formation also affects tolerance to osmotic, thermal stresses, and pH (Luo et al., 2015). In addition, type II secretion system, type III secretion system, and quorum sensing have been identified as major virulence factors (Wang et al., 2016; Zivanovic and Walcott, 2017; Zhang et al., 2018; Kan et al., 2023). It is also reported that the ferric uptake regulator, YggS family pyridoxal phosphate-dependent enzymes, pyridoxal phosphate-dependent aminotransferases, glycerol-3-phosphate dehydrogenases, and 2, 3-bisphosphoglycerate-dependent phosphoglycerate mutases, and the nitrogen regulator are associated with virulence in *Ac* (Liu et al., 2019; Kim et al., 2021; Lee et al., 2021; Lee et al., 2022b; Wang et al., 2022; Heo et al., 2023; Liu et al., 2023). However, these well-characterized factors/mechanisms involved are insufficient to comprehensively understand the virulence mechanisms of *Ac*. Therefore, further studies are required to elucidate the *Ac*’s virulence factors and related mechanisms in detail.

Glucose-6-phosphate isomerase (GPI), also known as phosphoglucose isomerase (PGI), is one of the key enzymes in both eukaryotic and prokaryotic organisms (Achari et al., 1981). It facilitates the reversible transformation of glucose-6-phosphate into fructose-6-phosphate within a complex network of carbon metabolic pathways, encompassing glycolysis/gluconeogenesis, pentose sugar, an amino sugar, and nucleotide sugar metabolisms (Achari et al., 1981; Zhang et al., 2015; Kim et al., 2020b). GPI is mostly regarded as a moonlighting protein due to its multifunctional role in diverse cellular activities, including sugar interconversion, serine proteinase inhibition, and mediation of differentiation (Jeffery et al., 2000; Angira et al., 2020). It is also associated with cell wall biosynthesis, contributing to virulence in *Aspergillus fumigatus* (Zhou et al., 2022). In *Xanthomonas campestris* pv. *citri*, a causal agent of citrus canker, GPI is essential for pathogenicity (Tung and Kuo, 1999). It is also reported that cryptococcal phosphoglucose isomerase is required for virulence factor production, cell wall integrity, and stress resistance (Zhang et al., 2015). Although these studies implicate that GPI is closely associated with bacterial virulence, sugar metabolism, and cell wall functions, the functions of GPI in *Ac* have not been documented to date.

This study reports the putative glucose-6-phosphate isomerase (GpiAc) functions in the group II *Ac* strain KACC17005 of *Ac*. To characterize the roles of GpiAc, the virulence and growth ability of *gpiAc:Tn5*, a *gpiAc-*defective mutant, were compared to the wild-type stains. To postulate the cellular mechanisms associated with GpiAc virulence, a label-free, shot-gun comparative proteomic analysis was conducted. To further confirm the pleiotropic functions of GpiAc, we investigated its involvement in biofilm formation, twitching halo, and tolerance to osmotic and alkali stresses. Our observation revealed that GpiAc is involved in diverse cellular mechanisms and virulence in *Ac*.

## Materials and methods

### Bacterial strains and growth conditions

The completely sequenced *Ac* group II strain KACC17005 was used as a wild-type strain (Park et al., 2017). *Ac* strains were grown in TSB (Tryptic Soy Broth Soybean-Casein Digested, 30g/L) and M9 (47.7 mM Na_2_HPO_4_·7H_2_O, 22 mM KH_2_PO_4_, 8.6 mM NaCl, 18.7 mM NH_4_Cl, 2 mM MgSO_4_, 0.1 mM CaCl_2_, 20 ml of 20% glucose in 1L) at 28°C. For gene cloning and plasmid construct formation, the DH5ɑ strain was used. The EC100D strain was used to identify the Tn5-inserted gene. *E. coli* strains were grown in LB medium (Luria Bertani; 1% tryptone, 0.5% yeast extract, and 1% NaCl) at 37°C. For selection, media was supplemented with appropriate antibiotics at the final concentrations: rifampicin, 50 μg/ml; kanamycin, 50 μg/ml; gentamycin, 10 μg/ml; and ampicillin, 100 μg/ml.

### Selection of *gpiAc:Tn5* and generation of GpiAc complemented strain

To select the knockout mutant, the Tn5-insertional mutant library generated by EZ-Tn5™<R6Kγori/KAN-2> Insertion Kit (Lucigen, Middleton, WI, USA) was screened as described previously (Lee et al., 2022b; Heo et al., 2023). After selecting a virulence-deficient mutant, the gene interrupted by Tn5 was identified using the manufacturer’s protocol. We observed that the identified gene encodes a putative glucose 6-phosphate isomerase (Accession No. ATG97016); therefore, the mutant was named *gpiAc:Tn5*. To generate a construct for strain complementation, an open reading frame of *gpiAc* gene was amplified using *gpiAc-*specific primers (Forward primer: 5`-gtcgagatgacgatgcccgcccgcgt-3` and reverse primer: 5`-aagcttcagtggtggtggtggtggtggcgacggccctgggcgagccgcgccat-3`). The amplicon was ligated into pGEM-T easy vector (Promega, Madison, WI, USA), producing pGEM-*gpiAc* plasmid. After confirming the clone by Sanger sequencing, the plasmid was digested with *Hin*dIII and *Bam*HI, and the digested DNA fragment was recloned into the pBBR1-MCS5 vector, which is a broad host range vector containing LacZ promoter (Kovach et al., 1995), creating pMCS5-GpiAc plasmid. Next, the plasmid was introduced into *gpiAc:Tn5* by electroporation (Bio-Rad), generating the complemented strain, *gpiAc:Tn5*(GpiAc). The complemented strain was confirmed by PCR using *gpiAc*-specific primers. To remove the side effects caused by the pBBR1-MCS5, the vector was also introduced into *Ac* and *gpiAc:Tn5*, generating *Ac*(EV) and *gpiAc:Tn5*(EV), respectively. Bacterial strains and plasmids used in this study are shown in **Supplementary Table 1**.

### Virulence assay

*Citrullus lanatus* var. *vulgaris* line SBA provided by Partner Seed Company (Gimje, Korea) was used to test the virulence of *Ac* strains. Two different virulence assays were performed as follows. The germinated-seed inoculation method was first carried out, as reported previously (Lee et al., 2022b). Briefly, the germinated seeds were incubated in 10 mM MgCl_2_ containing approximately 10^6^ colony-forming units (CFUs)/mL of *Ac* strains for one hour at 22°C. Ten seeds were used per strain. The infected seeds were placed in an environmentally controlled room with 25 ± 1°C, 16/8 day/night photoperiod, and approximately 70% humidity (relative). The disease severity was measured for 7 days on 0~2 scales (Lee et al., 2021). This assay was conducted at least four times with ten biological replicates per strain.


$$Disease\;Index:\frac{Normal\left(Plant_{n}\right)*0+Spot\left(Plant_{n}\right)*1+Wilt\left(Plant_{n}\right)*2}{Total\left(Plant_{n}\right)}$$

The leaf infiltration was performed following a previously established protocol (Kim et al., 2020a). Four true leaves stages were infiltrated using needless syringes. The final concentration of inoculum was approximately 10^5^ CFU/mL. Bacterial suspensions were infiltrated through the underside of the second true leaves. Two discs were obtained using cork borers (0.4 cm in diameter) and ground. The ground samples were serially diluted, dotted onto TSA plates, and viable colonies were counted. These measurements were carried out in two-day intervals for 8 days after inoculation. At least four independent experiments were carried out with three biological replicates per strain.

### Growth assay

*Ac* strains were adjusted to an OD_600nm_ of 0.3 (approximately 10^8^ CFU/mL), diluted to 10^5^ CFU/mL with TSB media, and incubated at 28°C in a shaking incubator for 84 hours. The growth of each strain was measured every 12 hours. Three independent experiments with three biological replicates were conducted. In the M9 medium, 0.4% glucose or fructose was used. The bacterial suspension was placed in an M9 medium containing each carbon source. The final bacterial concentration was an OD_600nm_ of 0.05 and the growth was determined every 24 hours for 10 days at 28°C in a shaking incubator. Each strain with three biological replicates was used, and three independent experiments were carried out.

### Proteomics analysis

A label-free proteomic analysis was carried out using previously established protocols (Lee et al., 2022a; Heo et al., 2023). *Ac* and *gpiAc:Tn5* strains with three biological replicates (6 samples) were used for the comparative analysis. Concisely, *Ac* strains incubated in TSB were harvested at an OD_600nm_ of 0.6. The harvested bacterial cells were resuspended into the lysis buffer (6M guanidine HCl, 10 mM dithiothreitol, 50 mM Tris-HCl pH 7.8). The cell suspension was disrupted by sonication, and the concentration of total soluble proteins was measured using a BCA kit (Thermo Fisher Scientific, Rockford, IL, USA). The samples were digested by trypsin to generate peptides. For the liquid chromatography followed by tandem mass spectrometry analysis (LC-MS/MS), one μg of the samples was analyzed by split-free nano-LC (EASY-nLC II; Thermo Fisher Scientific, Bremen, Germany) linked to the LTQ Velos Pro instrument (Thermo Fisher Scientific). The samples were separated in the column with MAGIF C18AQ 200A (Michrom BioResources, Auburn, CA, USA). Six data-dependent MS/MS scans were conducted to obtain the full MS spectra. The mass spectrometry proteomics data have been deposited to the ProteomeXchange Consortium via the PRIDE (Perez-Riverol et al., 2022) partner repository with the dataset identifier PXD042560.

The previously established method was followed for the identification and quantification of the proteins/peptides (Lee et al., 2022b). The LC-MS/MS-generated raw data were analyzed by Thermo proteome discoverer (ver. 1.3.0.399) with SEQUEST search algorithm. The genome information of the group II strain KACC17005 (Accession No. CP023687) was used to search for spectra information. The target-decoy strategy was used to improve credibility in this study (Elias and Gygi, 2007). The identified proteins were imported again into Scaffold 4 (Proteomic software, Portland, OR, USA) for the comparative proteome analysis. The peptide spectra match (PSM) (Choi et al., 2008) was used for comparative values. PSM values from individual proteins were normalized against the total PSMs in the sample. The mean value from the three biological replicates was used for identifying differentially abundant proteins (more than 2-fold) between *Ac* and *gpiAc:Tn5*. A student’s t-test (P<0.05) was conducted for statistical analysis. The differentially abundant proteins were categorized using a cluster of orthologous groups (COG) analysis (Tatusov et al., 2000).

### Biofilm formation assay

Biofilm formation assay was carried out as described previously (Lee et al., 2022b). Briefly, *Ac* strains grown in TSA were resuspended to an OD_600nm_ of 0.3 (approximately 10^8^ CFU/mL) and diluted to the final concentration (10^5^ CFU/mL) with TSB. Next, 190 μl of the suspension was placed into the 96-well polyvinyl chloride plate and incubated for two days at 28°C. The bacterial cells forming biofilm were stained with the crystal violet solution (0.1%) and dissolved in 95% ethanol. Biofilm formation was determined using the Spectramax 190 microplate reader (Molecular Devices, Sunnyvale, CA, USA) at 590nm. Twenty biological replicates of each strain were used in at least three independent experiments.

### Twitching halo assay

The twitching halo motility assay was performed following the previous study (Lee et al., 2022b). Bacterial cells were resuspended in water to an OD_600nm_ of 0.3 (approximately 10^8^ CFU/mL) and diluted to adjust the concentration to 10^6^ CFU/mL. The five μl bacterial suspension was dotted on the semi-solid (0.5% agar) TSA plates. Dotted samples were incubated at 28°C for two days. The colony size and twitching halo diameter were measured using the stereoscopic microscope LEICA M205 C (LEICA Wetzlar, Germany). Three replicates of each strain were used in the assay, and at least four independent experiments were carried out.

### Stress tolerance assay

For the alkali and osmotic stress tolerance assay, bacterial cells were adjusted to an OD_600nm_ of 0.3 (approximately 10^8^ CFU/mL) and serially diluted 10-fold from 10^0^ to 10^5^ with water. The diluted samples were dotted onto TSA containing sodium chloride (NaCl, 1.5% or 2%) or TSA (pH 9 and 10). The untreated TSA was used as a control. For lysozyme treatment, bacterial cells were adjusted to an OD_600nm_ of 0.3. Each strain was treated with 6 μg/mL of lysozyme for two hours in TSB. Untreated TSB was used as a control. After the treatment, the bacterial suspension was serially diluted and dotted onto TSA. The survivability of each condition was established using the ratio of viable cell numbers from the treated samples to untreated samples. At least three independent experiments were conducted with the three biological replicates for each stress condition.

### Statistical analysis

The statistical significance of quantitative data was analyzed by student’s t-test and one-way analysis of variance with Turkey HSD^ab^ using SPSS 12.0K (Chicago, IL, USA). A p-value (< 0.05) indicated a statistically significant difference.

## Results

### GpiAc is indispensable for virulence of *Ac*

**Figure 1:**
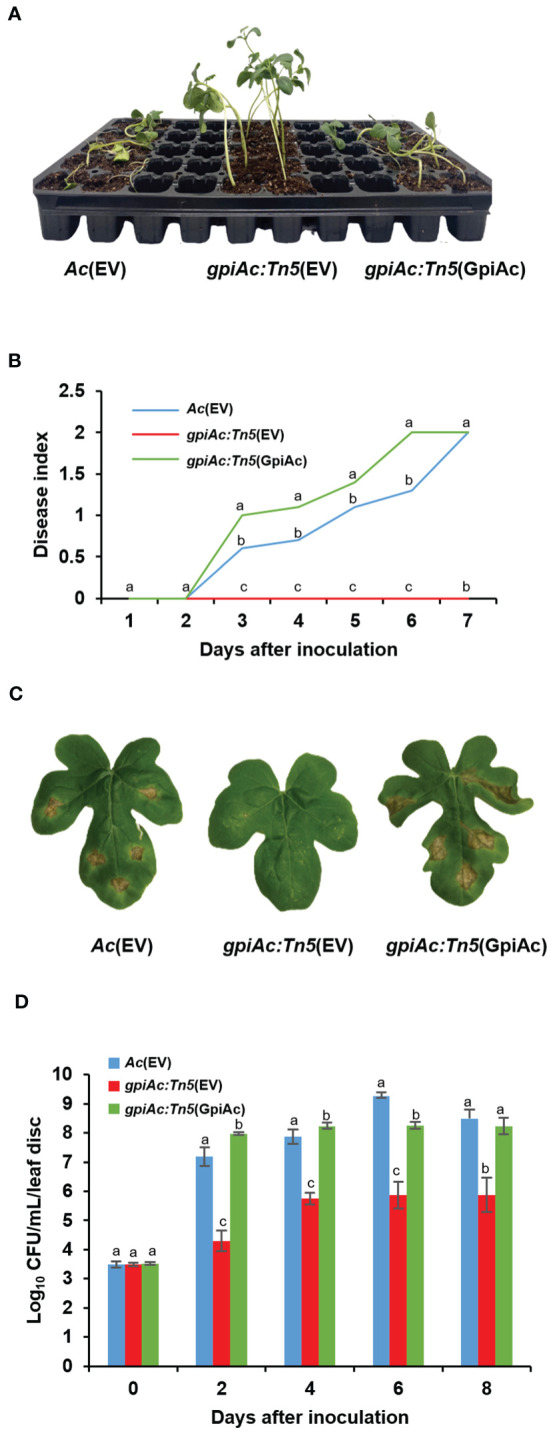
Virulence assay for *Ac*(EV), *gpiAc:Tn5*(EV), and *gpiAc:Tn5*(GpiAc). **(A)** The photograph was captured seven days after inoculation from the germinated seed inoculation. **(B)** Disease index variation during seven days in the seed-germinated inoculation. The disease index: [(numbers of plants with no symptoms) × 0 + (numbers of spotted plants) × 1 + (numbers of wilted plants) × 2]/Total (numbers of plants). **(C)** The photographs were captured 8 days after inoculation from the leaf infiltration. **(D)** The living bacterial population in infiltrated leaves for 8 days using the colony counting method. All strains were inoculated into the leaves of a two-week-old watermelon using the syringe without a needle. Different letters on the error bars (standard errors of means) represent the statistical difference by ANOVA (p<0.05) with Turkey HSD^ab^. At least four independent experiments were carried out and displayed similar patterns.

We identified a virulence-deficient mutant during the screen of a Tn5-insertional mutant library using the group II strain KACC17005. A putative glucose 6-phosphate isomerase in *Ac* (GpiAc) (Accession No. ATG97016) gene was observed to be disrupted by Tn5 in the virulence-deficient mutant. To examine whether GpiAc contributes to virulence, two virulence assays, germinated-seed inoculation and leaf infiltration, were conducted in three strains; *Ac*(EV), *gpiAc:Tn5*(EV), and *gpiAc:Tn5*(GpiAc). *Ac*(EV) and *gpiAc:Tn5*(EV) are the wild-type and *gpiAc* null mutant strains containing the empty vector, respectively. *gpiAc:Tn5*(GpiAc) is the mutant carrying the open reading frame of the *gpiAc* gene on the pBBR1-MCS5 vector. In the germinated-seed inoculation assay, severe disease symptoms were observed in *Ac*(EV), which continued to increase up to a disease index value of 2 at 7 days after inoculation (DAI) (**Figures 1A, B**). On the other hand, no symptoms were found in *gpiAc:Tn5*(EV), and the disease index remained at 0 at 7 DAI. The virulence of the complemented stains, *gpiAc:Tn5*(GpiAc), was restored, and its disease index value reached 2 at 7 DAI, demonstrating no positional effect by Tn5 insertion. The outcomes of the leaf infiltration were comparable to the findings obtained from the germinated-seed inoculation. In *Ac*(EV) and *gpiAc:Tn5*(GpiAc), typical disease symptoms, such as dark leaves and wilting, were confirmed (**Figure 1C**). However, the leaf infiltrated by *gpiAc:Tn5*(EV) showed no symptoms. Further, we evaluated the bacterial growth in watermelon leaves infected with three strains (**Figure 1D**). The growth of *gpiAc:Tn5*(EV) was significantly lower than that of *Ac*(EV) and *gpiAc:Tn5*(GpiAc) at 2, 4, 6, and 8 DAI. The *gpiAc:Tn5*(EV) population was comparable to *gpiAc:Tn5*(GpiAc). These results demonstrated that GpiAc is indispensable for the virulence of *Ac*.

### GpiAc is not associated with multiplication in *Ac*

**Figure 2:**
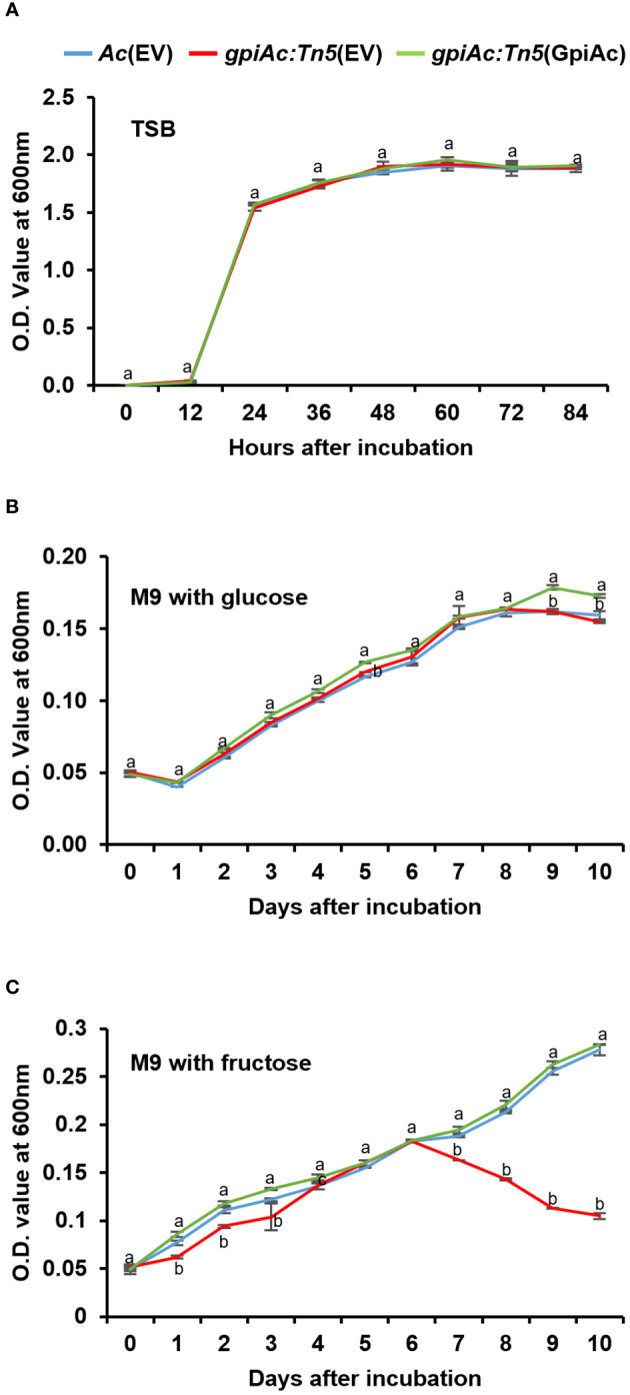
The bacterial growth curve in rich and minimal media with sole carbon source. Bacterial growth measurement in **(A)** TSB, M9 minimal supplemented with 0.4% (final concentration) of **(B)** glucose and **(C)** fructose as a sole carbon source. The population of bacterial strains was measured at OD_600nm_ using a UV spectrophotometer with **(A)** 12h intervals and **(B, C)** 24h intervals. Different letters indicate statistically significant differences by ANOVA (p<0.05) with Turkey HSD^ab^. At least three independent experiments were conducted, showing similar patterns.

The bacterial growth and virulence of *gpiAc:Tn5*(EV) were decreased in both virulence assays compared with those of *Ac*(EV) and *gpiAc:Tn5*(GpiAc) (**Figure 1**). To determine whether the reduction in growth and virulence in watermelon was due to the difference in the multiplication of the mutant, we investigated the bacterial growth curves of three strains, *Ac*(EV), *gpiAc:Tn5*(EV), and *gpiAc:Tn5*(GpiAc), in TSB (the nutrient-rich condition). All three strains showed similar growth patterns (**Figure 2A**). *Ac* strains reached the log phase 12 hours after incubation and the stationary phase 24 hours after incubation. There was no statistically significant difference in the growth curves during the observation period among the three strains.

### GpiAc is involved in fructose utilization in *Ac*

Since GpiAc catalyzes the conversion of glucose-6-phosphate to fructose-6-phosphate, we sought to determine whether GpiAc affects carbon source utilization by examining growth differences in M9 minimal medium supplemented with glucose or fructose for 10 days. The growth of the three strains was comparable in the M9 medium containing glucose (**Figure 2B**). Interestingly, the growth pattern of *gpiAc:Tn5*(EV) differed from the wild-type and complemented strains in the M9 medium supplemented with fructose (**Figure 2C**). All three strains showed similar growth patterns until 6 DAI. However, the growth of *gpiAc:Tn5*(EV) was significantly reduced from 7 DAI to the end of the incubation period. These results indicate that GpiAc is involved in fructose utilization in *Ac*.

### Proteomic analysis

**Figure 3:**
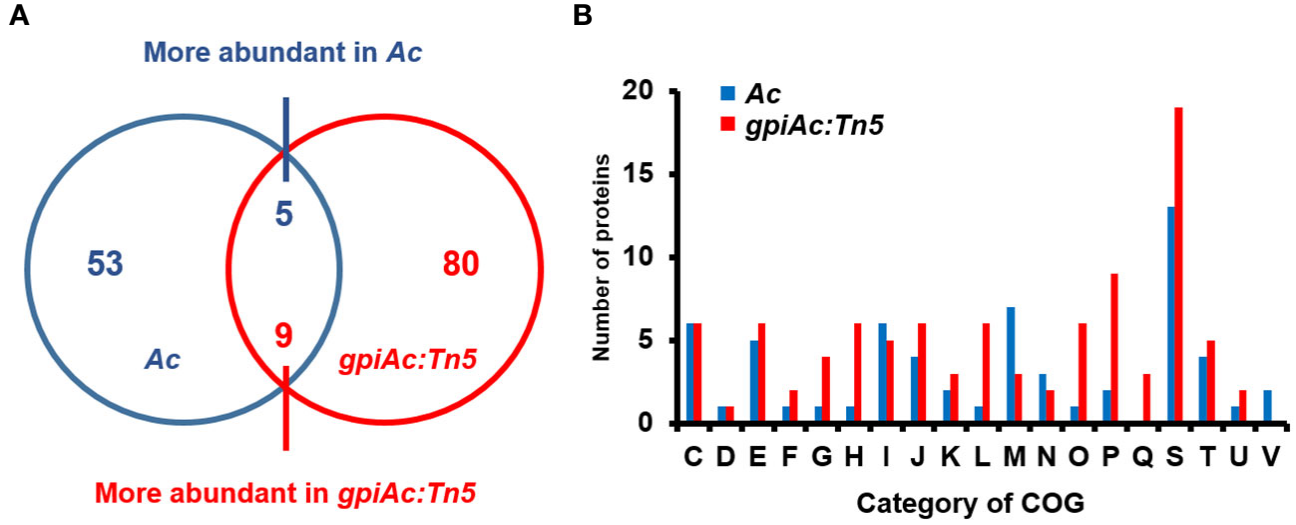
Comparative proteomic analysis of *Ac* and *gpiAc:Tn5*. **(A)** Venn diagram displaying the number of the differentially (over two-fold) abundant proteins from the proteomic analysis. **(B)** Fifty-three and 80 proteins were uniquely identified, and 5 and 9 proteins were more abundant in *Ac* and *gpiAc:Tn5* strains, respectively. **(B)** A cluster orthologous group (COG) analysis of proteins from the comparative analysis. C, Energy production and conversion; D, Cell cycle control and mitosis; E, Amino acid metabolism and transport; F, Nucleotide metabolism and transport; G, Carbohydrate metabolism and transport; H, Coenzyme metabolism; I, Lipid metabolism; J, Translation; K, Transcription; L, Replication and repair; M, Cell wall/membrane/envelop biogenesis; N, Cell motility; O, Post-translational modification, protein turnover, chaperone functions; P, Inorganic ion transport and metabolism; Q, Secondary structure; S, Function unknown; T, Signal transduction; U, Intracellular trafficking and secretion; V, Defense mechanisms.

We ascertained that GpiAc is indispensable for virulence and the growth in M9 with fructose. A previous study revealed that BdpmAc is associated with fructose metabolism as well as other phenotypes, including biofilm formation and tolerance to osmotic stress (Lee et al., 2022b). Therefore, to postulate the biological mechanisms affected by GpiAc, comparative proteomic analysis based on the label-free shotgun approach was performed using *Ac* and *gpiAc:Tn5* strains. In the three biological replicates of the wild-type strain, 1105, 1108, and 1093 proteins were identified from 59582, 59503, and 59560 peptide spectra matches (PSMs), respectively (**Supplementary Table 2**). In the *gpiAc:Tn5* strain, 1161, 1149, and 1139 proteins were detected from 57989, 58005, and 58007 PSMs, respectively. Among the proteins, 1046 and 1069 proteins were commonly found in the three biological replicates of *Ac* and *gpiAc:Tn5* strains, respectively. The comparative analysis of commonly identified proteins showed that 53 and 80 proteins were uniquely detected, and 5 and 9 proteins were more abundant (over 2-fold) in *Ac* and *gpiAc:Tn5*, respectively (**Figure 3A**). The differentially abundant proteins were categorized by cluster of orthologous groups (COG) analysis (**Figure 3B**, **Supplementary Tables 3**, **4**). Proteins belonging to group I (Lipid metabolism), M (Cell wall/membrane/envelope biogenesis), N (Cell motility), and V (Defense mechanisms) were markedly abundant in the wild-type strain. Proteins categorized in group G (Carbohydrate metabolism and transport), H (Coenzyme metabolism), L (Replication), O (Post-translational modification, protein turnover, chaperone functions), P (Inorganic ion transport and metabolism), and Q (Secondary structure) were significantly detected in the mutant. Specifically, the abundance of virulence-related proteins was altered, including phospholipase, two-component system response regulators, DNA-binding response regulators, and bifunctional diguanylate cyclase/phosphodiesterases. In addition, proteins related to bacterial motility were identified, such as flagella basal body protein FliL and three methyl-accepting chemotaxis proteins. Moreover, diverse proteins (TonB-dependent receptor, protein TolA, UDP-3-O-[3-hydroxymyristoyl] N-acetylglucosamine deacetylase, N-acetyltransferase, and transglycosylase) involved in cell wall/membrane function, were detected in our study. The comparative proteomic analysis reveals that GpiAc is involved in virulence, bacterial motility, and cell wall/membrane functions.

### *gpiAc:Tn5* reduces biofilm formation

**Figure 4:**
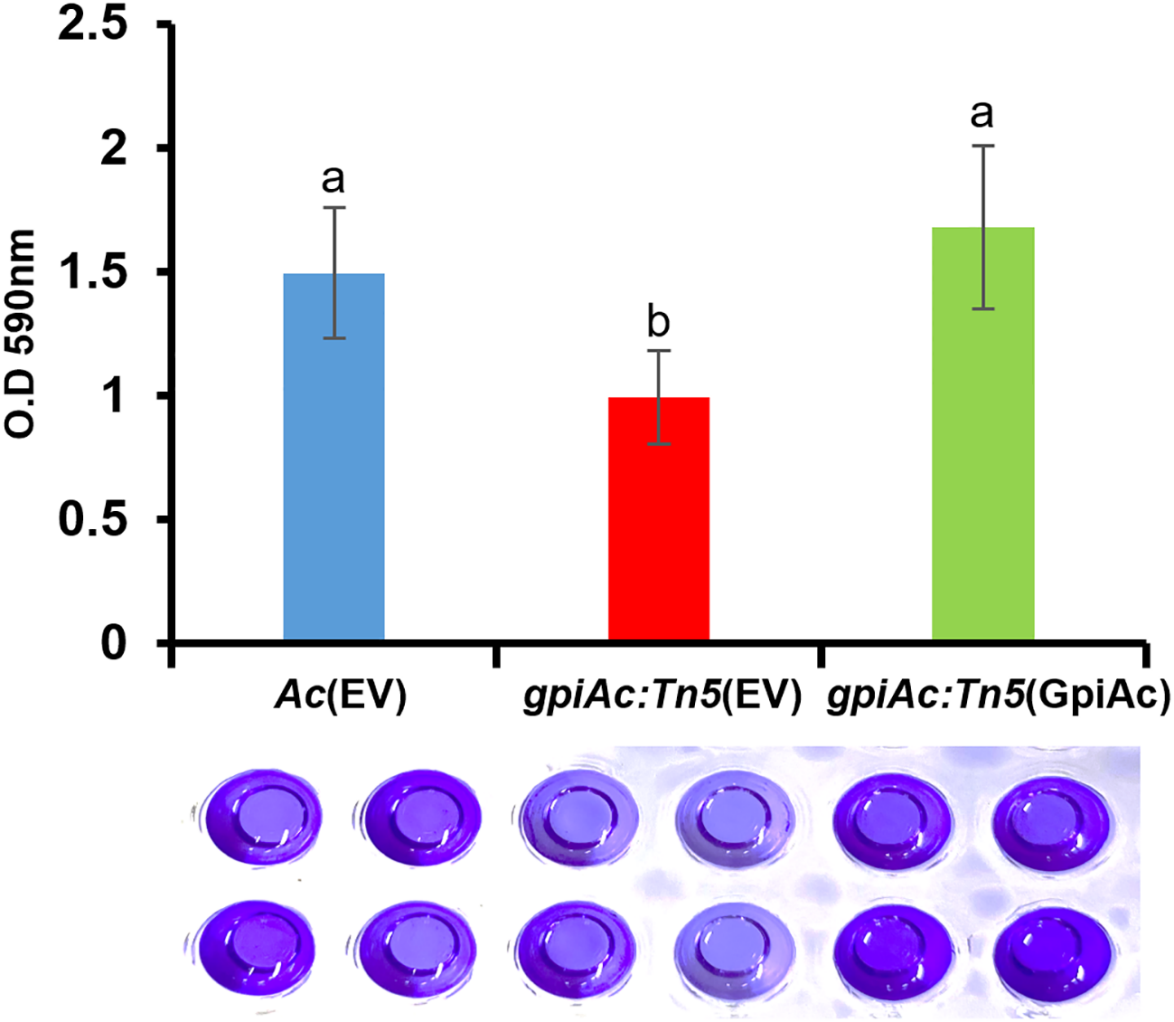
Biofilm formation from *Ac*(EV), *gpiAc:Tn5*(EV), and *gpiAc:Tn5*(GpiAc). *Ac* strains were incubated in TSB on polyvinyl chloride 96-well plates, and biofilm formation was quantified after two days of incubation. The amount of biofilm formed by three strains. Different letters on error bars represent the statistical difference by ANOVA (p<0.05) with Turkey HSD^ab^. A photograph of biofilm stained with 0.1% crystal violet and eluted with 95% ethanol was captured. Three independent experiments with twenty biological replicates were carried out, and all experiments exhibited similar patterns.

Plant pathogenic bacteria form biofilms for protection against external or environmental stresses, which is an important pathogenic factor (Davey and O’Toole, 2000). Our comparative proteomics analysis suggests that GpiAc may affect the cell wall/membrane functions required for biofilm formation. A previous study reported that glucose-6-phosphate, a putative substrate for GpiAc, is involved in biofilm formation in *Pseudomonas aeruginosa* (Park et al., 2021). Therefore, we evaluated the ability of biofilm formation in *Ac*(EV), *gpiAc:Tn5*(EV), and *gpiAc:Tn5*(GpiAc) using the 96-well PVC plate assay. We observed that the biofilm formation ability of *gpiAc:Tn5*(EV) was significantly decreased compared to *Ac*(EV) (**Figure 4**). The complemented strains, *gpiAc:Tn5*(GpiAc), restored the biofilm formation function to *Ac*(EV) level. These results highlight that GpiAc is associated with biofilm formation.

### *gpiAc:Tn5* reduces twitching motility

**Figure 5:**
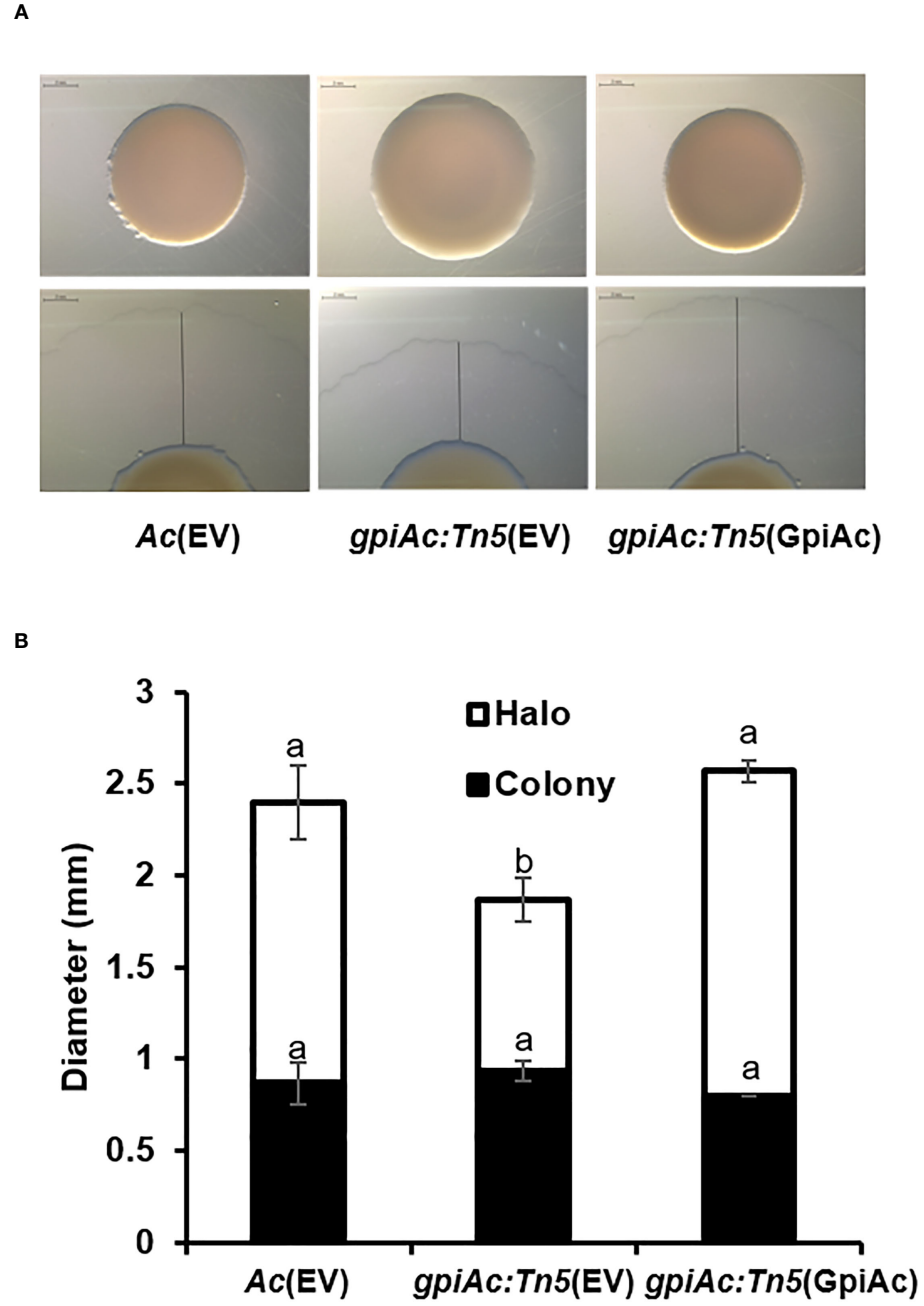
Twitching halo production in *Ac*(EV), *gpiAc:Tn5*(EV), and *gpiAc:Tn5*(GpiAc). Five μL of bacterial suspension (10^8^ CFU/mL) was dotted onto TSA medium containing 0.5% agar and incubated for two days. **(A)** Images from twitching motility assay. The black line indicates the halo size. The scale bar is 2 mm. **(B)** Measurement of the sizes from the colony and twitching halo. The black and white bars indicate the diameter of the colony and twitching halo, respectively. The error bars indicate standard deviations. Different letters indicate statistically significant differences by ANOVA (p<0.05) with Turkey HSD^ab^. Four independent experiments were conducted, all of which showed similar patterns.

Like other bacteria, *Ac* possesses two types of motilities; pili- and flagella-dependent, while the group II strains, including KACC17005, can only show pili-dependent motility in *in vitro* conditions (Bahar et al., 2010). In addition, diverse proteins associated with bacterial movement were identified in the COG classification. Therefore, the twitching motility, which is pili-dependent motility, was examined in the TSA plate with 0.5% agar, and the colony and twitching halos sizes were measured from the three strains (**Figure 5**). The colony size of *gpiAc:Tn5*(EV) was not different from those of *Ac*(EV) and *gpiAc:Tn5*(GpiAc). On the other hand, the twitching halo size of *gpiAc:Tn5*(EV) was significantly reduced (0.93 cm) compared to that (1.5 cm) of the wild-type strain. The production of the twitching halo was reestablished (1.7 cm) in *gpiAc:Tn5*(GpiAc). There was no statistical difference in the sizes of *gpiAc:Tn5*(GpiAc) and *Ac*(EV). These results indicate that GpiAc is associated with twitching motility in *Ac*.

### GpiAc is involved in stress tolerance

**Figure 6:**
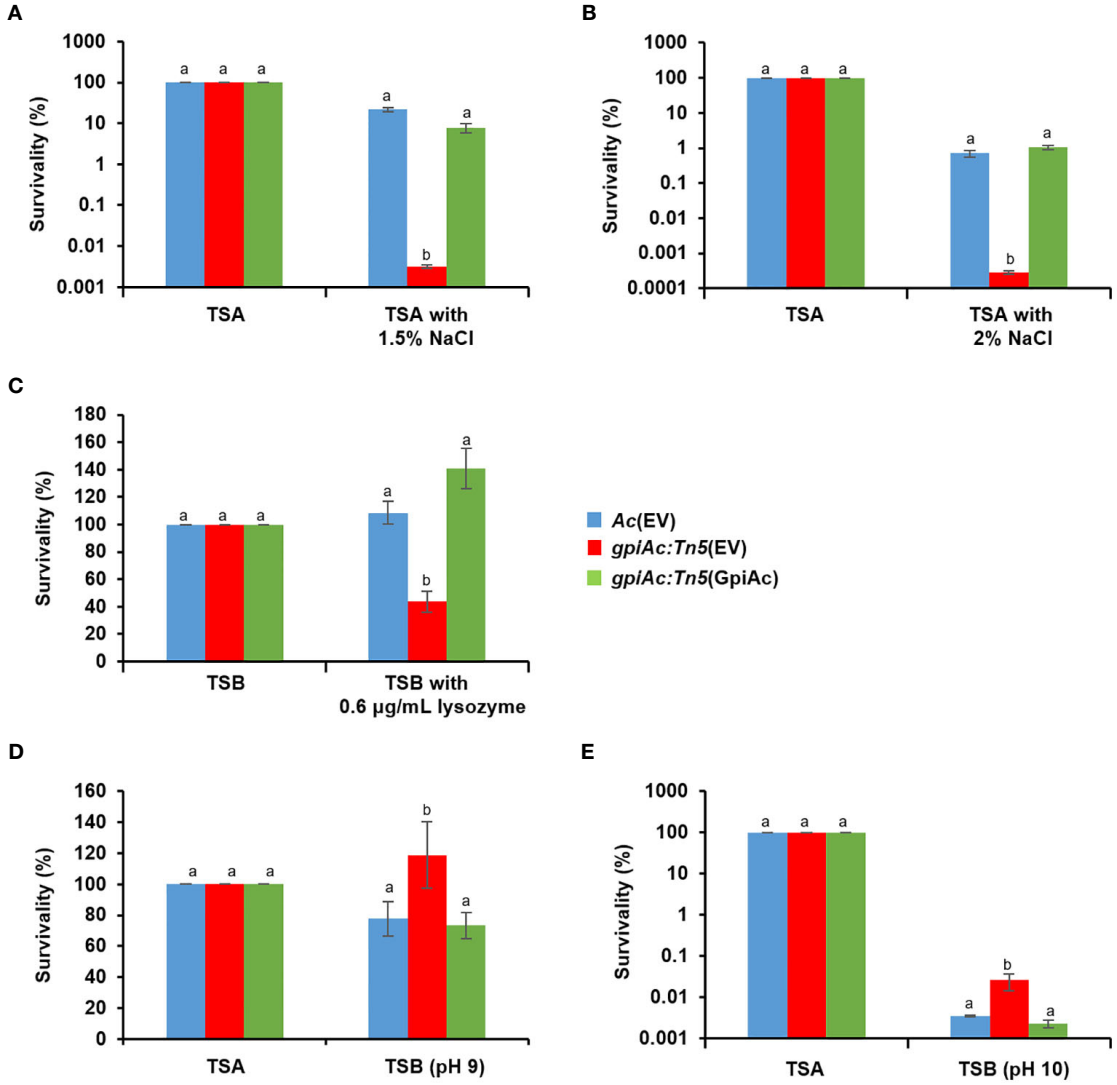
Tolerance assay for osmotic stress, lysozyme, and pH treatment. Tolerance was determined in the rich media (TSA or TSB) in the presence of **(A)** 1.5% NaCl, **(B)** 2% NaCl, **(C)** 0.6 μg/mL lysozyme, and **(D)** pH 9, and **(E)** pH 10 treatment. The rich media without stress factors were used as a negative control. The numbers of viable cell numbers were established by the colony counting method. The survivability was calculated based on the ratio of the viable cell numbers from the stress conditions to the control. Different letters indicate statistically significant differences by ANOVA (p<0.05) with Turkey HSD^ab^. At least three independent experiments were carried out exhibiting similar patterns.

The comparative proteomic analysis identified several proteins related to the cell wall/membrane. Therefore, we further investigated the involvement of GpiAc in membrane/wall integrity and tolerance to various stresses in *Ac*(EV), *gpiAc:Tn5*(EV), and *gpiAc:Tn5*(GpiAc). Firstly, tolerance to osmotic stress was evaluated using NaCl (**Figures 6A, B**). The three strains were exposed to TSA media supplemented with 1.5% and 2% NaCl. *gpiAc:Tn5*(EV) showed survivability of 0.003% and 0.0003% in 1.5% and 2% NaCl, respectively, which was dramatically reduced compared to the survivability of 21.8% and 0.7% of *Ac*(EV). In addition, *gpiAc:Tn5*(GpiAc) showed survivability similar to wild-type stains (8% and 1%). Next, *Ac* strains were incubated in TSB with 0.6 μg/mL lysozyme for 2 hours (**Figure 6C**). The results were similar to the osmotic stress assay. The survivability in *gpiAc:Tn5*(EV) was significantly reduced with lysozyme treatment. Precisely, *Ac*(EV), *gpiAc:Tn5*(EV), and *gpiAc:Tn5*(GpiAc) showed 109%, 44%, and 141% survivability, respectively. On the other hand, in the alkali condition, the mutant exhibited opposite survivability patterns to other stresses. In the pH 9 condition, the survivability of *gpiAc:Tn5*(EV) (118.9%) was significantly enhanced compared to that of *Ac*(EV) (77.8%) (**Figure 6D**). *gpiAc:Tn5*(GpiAc) displayed comparable survivability (73.3%) to the wild-type strain. The survivability patterns of strains incubated at pH 10 were similar to those at pH 9 (**Figure 6E**). However, in the acidic condition (pH 5), the patterns of the three strains were similar (data not shown). Taken together, these results indicate that GpiAc is associated with environmental stress tolerance.

## Discussion

Previously, it has been shown that glucose-6-phosphate isomerases (GPIs) are involved in bacterial virulence in the Gram-negative bacterium *X. oryzae* pv. *oryzicola* and the Gram-positive bacterium, *Cryptococcus neoformans* (Zhang et al., 2015; Guo et al., 2017). In addition, GPIs-related mechanisms, glycolysis and gluconeogenesis, are also crucial for virulence in various plant and animal pathogenic bacteria, including *Staphylococcus aureus, Erwinia amylovora, Mycobacterium marinum*, and *X. axonopodis* pv. *glycines* (Vitko et al., 2015; Tong et al., 2016; Klee et al., 2019; Guo et al., 2020). Consistent with these observations, we demonstrate that GpiAc is indispensable for virulence in *Ac* using germinated seed inoculation and leaf infiltration assays. Notably, the growth pattern of *gpiAc:Tn5*(EV) in TSB was not different from *Ac*(EV). These results indicate that the reduced virulence in *gpiAc:Tn5*(EV) is not due to bacterial multiplication. In addition, both *Ac*(EV) and *gpiAc:Tn5*(EV) display similar patterns in M9 containing glucose, pointing out that *gpiAc:Tn5*(EV) is not an auxotroph, and GpiAc is not related to the production of primary metabolites.

GPI is a reversible and essential enzyme in glycolysis/gluconeogenesis. Glucose-6-phosphate and fructose-6-phosphate are substrates for GPI in glycolysis and gluconeogenesis pathways, respectively (Hers and Hue, 1983). Interestingly, *gpiAc:Tn5*(EV) could grow in an M9 medium supplemented with glucose. However, any GPI-homologs except GpiAc were not annotated in the KACC17005 genome (Park et al., 2017), suggesting an alternative pathway for glucose utilization in this strain. The Entner-Doudoroff pathway has been reported as another glucose catabolism pathway in gram-negative bacteria (Kersters and De Ley, 1968; Conway, 1992). Specifically, two enzymes, phosphogluconate dehydratase and keto-deoxy-phosphogluconate aldolase, are reported to be crucial for the Entner-Doudoroff pathway (Conway, 1992). The strain KACC17005 contains two genes, ATG92716 and ATG92717, encoding a putative phosphogluconate dehydratase and a putative keto-deoxy-phosphogluconate aldolase, respectively (Park et al., 2017). Therefore, it is postulated that *gpiAc:Tn5*(EV) likely employs the Entner-Doudoroff pathway and can use glucose as the sole carbon source in the M9 medium. However, it is not completely ruled out that the strain KACC17005 carries genes for GPI function. In contrast to the glucose metabolism pathways, an alternative fructose metabolism pathway has not been documented in bacteria. Our results, in which *gpiAc:Tn5*(EV) did not grow well in M9 supplemented with fructose, also indicate no alternative fructose pathway in *Ac*. Altogether, we speculated that glycolysis can proceed without GpiAc, but gluconeogenesis is attenuated in the mutant.

In addition to the interconversion of sugar, GPI is associated with diverse functions as a neurotrophic factor, an autocrine motility factor, and a differentiation/maturation mediator in the eukaryotic organism (Sriram et al., 2005). However, these activities are not conserved in all organisms. In *Cryptococcus neoformans*, the GPI mutant exhibits increased sensitivity to cell wall-damaging agents and osmotic stresses (Zhang et al., 2015). Moreover, GPI is required for extracellular polysaccharide (EPS) biosynthesis, cell motility, and full virulence of *X. oryzae* pv. *oryzicola* in rice (Guo et al., 2017). In agreement with these previous reports, our comparative proteomic analysis implicates that GpiAc is associated with various biological mechanisms. Further, phenotypic observation substantiates the involvement of GpiAc in diverse functions, indicating that GpiAc shows a pleiotropic effect.

Bacterial cell wall/membrane/envelope are essential for biofilm formation as well as response to environmental stresses (Hews et al., 2019; Viljoen et al., 2020). The proteomic analysis identified diverse proteins associated with cell wall/membrane/envelope biosynthesis and its related mechanisms. Among these proteins, two alpha/beta hydrolases (ATG94531, ATG95002) were uniquely detected in *gpiAc:Tn5*. A previous study reported that the overexpression of alpha/beta hydrolase led to the reduction of biofilm formation and pathogenicity in *Pseudomonas fluorescens* and *Pectobacterium carotovorum*, respectively (Mei et al., 2010). In our study, biofilm formation was also reduced in *gpiAc:Tn5*(EV). In addition, biofilms are known as a major virulence factor of bacteria (Davey and O'Toole, 2000). Therefore, the function of GpiAc is associated with biofilm formation, contributing to virulence in *Ac*. Furthermore, we also demonstrated that the mutant lacking GpiAc exhibited reduced tolerance to osmotic stress and lysozyme treatment in the rich condition, in which the mutant showed similar growth patterns with the wild-type strain. In addition to the osmotic stresses, pH condition is also crucial for bacterial membrane homeostasis (Krulwich et al., 2011). The optimal pH condition for *Ac* is around neutral (pH), but the bacterium can grow in pH 5 ~ 9 (Luo et al., 2015). Our study showed that the tolerance to alkali conditions in the mutant was enhanced. It suggests that membrane integrity is affected by the absence of GpiAc in the mutant, resulting in the alteration of tolerance to alkali conditions. These results indicate that GipAc is related to cell wall/membrane integrity in *Ac*. Although the specific molecular mechanisms underlying the pleiotropic roles of GpiAc have to be elucidated, it is clear that GpiAc affects various phenotypes along with virulence.

Cell motility requires energy from carbon metabolism (Mitchell and Kogure, 2006). Previously, it was reported that genes encoding catalytic enzymes in the carbohydrate catabolic pathway are required for cell motility in plant pathogens (Guo et al., 2017). The comparative proteomic analysis identified cell motility-related proteins, including TolA protein (ATG93209), which was more abundant in the wild-type strain. Inactivation of TolA in *E. coli* resulted in a complete loss of motility as well as pathogenic attenuation (Morgan et al., 2014). In the nutrient-rich condition (TSB), the growth of *gpiAc:Tn5*(EV) was not impaired because the medium contained enough carbon sources for the survival and multiplication of the mutant. Surprisingly, the twitching halo size produced by *gpiAc:Tn5*(EV) was significantly reduced in the same nutrient conditions. These results also suggest that GpiAc possesses diverse roles in virulence as well as other phenotypic mechanisms in *Ac*.

In summary, we demonstrated that GpiAc, a putative glucose 6-phosphate isomerase, is not only involved in glycolysis/gluconeogenesis but also exerts a broad pleiotropic effect on bacterial virulence, biofilm formation, motility, and tolerance to various external stresses. Our study provides new insights into the functions of a glucose 6-phosphate isomerase exhibiting pleiotropic effects in *Ac*. Particularly, GpiAc could be used as a potential target to interfere with bacterial virulence by anti-virulence reagents to reduce the occurrence of BFB disease in watermelon.

## Data availability statement

The datasets presented in this study can be found in online repositories. The names of the repository/repositories and accession number(s) can be found in the article/**Supplementary Material**.

## Author contributions

LH: Data curation, Formal Analysis, Investigation, Methodology, Visualization, Writing – original draft. YH: Investigation, Methodology, Writing – review & editing. YC: Investigation, Methodology, Writing – review & editing. JC: Investigation, Methodology, Writing – review & editing. JL: Investigation, Methodology, Writing – review & editing. SH: Conceptualization, Data curation, Formal Analysis, Funding acquisition, Methodology, Project administration, Resources, Supervision, Visualization, Writing – review & editing, Writing – original draft.
