# Supplementary material for: A putative glucose 6-phosphate isomerase has pleiotropic functions on virulence and other mechanisms in Acidovorax citrulli
Source: Front Plant Sci. 2023 Nov 7;14:1275438. doi: 10.3389/fpls.2023.1275438 (PMC10664246; doi:10.3389/fpls.2023.1275438)
Supplement: Supplementary file 1 [file Table_1.docx]

| **Supplementary Table 1. Bacterial strains and plasmids used in this study** | | |
| --- | --- | --- |
| Strains or plasmids | Characteristic(s) | Source or reference |
| *Escherichia coli* |  |  |
| EC100D | For identifying Tn5-insertional sites | Epicentre |
| DH5α | For cloning | Promega |
|  |  |  |
| *Acidovorax citrulli* |  |  |
| KACC17005 | Wild-type, Rif^r^ | (Park et al., 2017) |
| *Ac*(EV) | Wild-type carrying the pBBR1-MCS5, Rif^r^, Gm^r^ | This study |
| *gpiAc:Tn5* | Knockout mutant, Tn5-inserted in *gpiAc* gene, Rif^r^, Kan^r^ | This study |
| *gpiAc:Tn5*(EV) | *gpiAc:Tn5* carrying the pBBR1-MCS5, Rif^r^, Kan^r^, Gm^r^ | This study |
| *gpiAc:Tn5*(GpiAc) | Complemented strain, *gpiAc:Tn5* carrying the MCS5-GpiAc, Rif^r^, Kan^r^, Gm^r^ | This study |
|  |  |  |
| Plasmids |  |  |
| pGem-T easy | T-A cloning vector, Amp^r^ | Promega |
| pGem-*gpiAc* | pGem-T easy vector ligating 1628 bp of *gpiAc* gene, Amp^r^ | This study |
| pBBR1-MCS5 | Broad-host-range vector, LacZ promoter, Gm^r^ | (Kovach *et al.*, 1995) |
| pMCS5-GpiAc | pBBR1-MCS5 carrying the *gpiAc* gene from pGem-*gpiAc* plasmid, Gm^r^ | This study |
| Rif^r^, Kan^r^, Gm^r^ and Amp^r^ represent resistance to Rifampicin, Kanamycin, Gentamycin, and Ampicillin, respectively. | | |
