# Supplementary material for: A putative glucose 6-phosphate isomerase has pleiotropic functions on virulence and other mechanisms in Acidovorax citrulli
Source: Front Plant Sci. 2023 Nov 7;14:1275438. doi: 10.3389/fpls.2023.1275438 (PMC10664246; doi:10.3389/fpls.2023.1275438)
Supplement: Supplementary file 2 [file Table_2.docx]

**Supplementary Table 2. Proteins and peptide spectral matches (PSM) between *Ac* and *gpiAc:Tn5***

| **Strain** | **1st** | | **2nd** | | | | **3rd** | | **shared proteins in 3 biological replicates** |
| --- | --- | --- | --- | --- | --- | --- | --- | --- | --- |
|  | **Protein** | **PSM** | | **Protein** | **PSM** | **Protein** | | **PSM** |  |
| *Ac* | 1,105 | 59,582 | | 1,108 | 59,593 | 1,093 | | 59,560 | 1,046 |
| *gpiAc:Tn5* | 1,161 | 57,989 | | 1,149 | 58,005 | 1,139 | | 58,007 | 1,069 |
